# Pulmonary artery vasodilators for treatment of pulmonary hypertension complicating fibrosing mediastinitis

**DOI:** 10.1016/j.rmcr.2024.102006

**Published:** 2024-03-16

**Authors:** Daniel Van Kalsbeek, Abedel Rahman Anani, Karim El-Kersh

**Affiliations:** University of Nebraska Medical Center, Omaha, NE, USA

**Keywords:** Fibrosing mediastinitis, Mediastinal fibrosis, Sclerosing mediastinitis, Pulmonary hypertension, Pulmonary vasodilators

## Abstract

Fibrosing mediastinitis (FM) is a heterogeneous disease characterized by sclerosing fibrosis of mediastinal structures. Pulmonary hypertension (PH) may complicate the course of the disease and can contribute significantly to the morbidity of FM. Due to the rarity and complexity of the disease, evidence-based guidelines are not currently available, and the optimal treatment approach is unknown. Management approaches should be individualized, and current techniques are often unsatisfactory. Here, we present two cases of PH complicating FM that were managed using pulmonary artery vasodilator therapy with excellent hemodynamic response.

## 1 Introduction

Fibrosing mediastinitis (FM) is a non-malignant fibroproliferative disease represented by sclerosing fibrosis of the mediastinum and related structures. It is a rare and complex disorder with multiple etiologies, various presentations, and limited treatment options [1,2]. Pulmonary hypertension (PH) is a well described complication of FM and is a leading cause of morbidity and mortality in this disease [1,3,4]. Due to the rareness of the disease and its heterogeneous nature, evidence-based treatment guidelines for PH caused by FM (PH-FM) do not exist and management must be highly individualized. Treatment of the underlying cause of FM is generally ineffective [1,3]. Therapeutic approaches for PH-FM include supplemental oxygen, diuretics, surgical or percutaneous vascular interventions, and/or pharmacologic pulmonary vasodilators [1,2]. Vasodilator therapy is an appealing treatment for selected cases of precapillary PH-FM because invasive vascular repair carries a high complication and failure rate in this disease [2,5,6]. However, given the paucity of data, the effectiveness of vasodilatory therapy in PH-FM is unclear and patients must be thoroughly evaluated for appropriateness of this therapy [1,4]. Here, we present two cases of PH-FM that were managed successfully with pulmonary vasodilator therapy.

## 2 Case 1

**Fig. 1:**
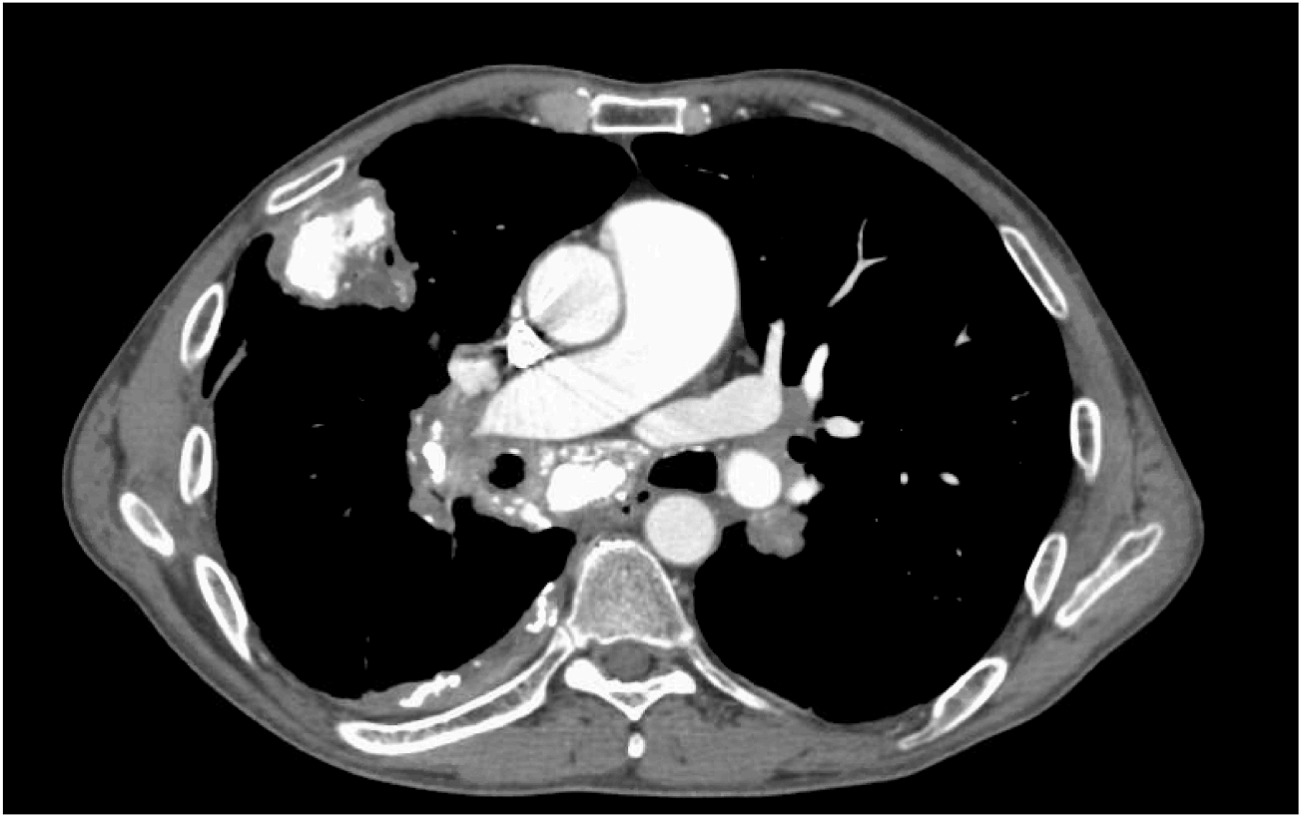
Axial view of computed tomography pulmonary angiography showing severe stenosis of the distal right pulmonary artery. Also in view is calcified mediastinal fibrosis, a calcified pulmonary mass, and a calcified pleural plaque.

**Fig. 2:**
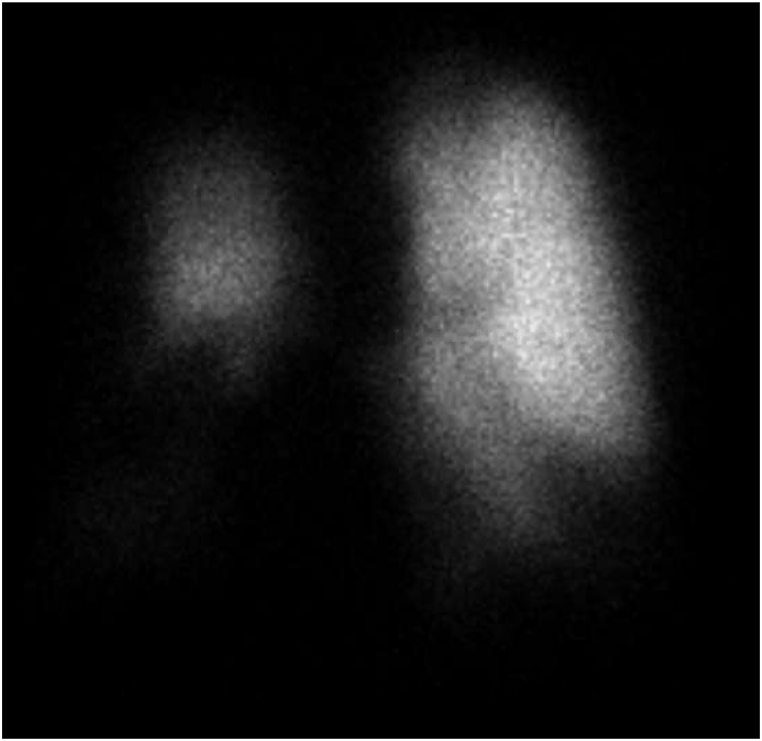
Anterior projection perfusion scan showing perfusion defects in the region of the right middle and right lower lung zones.

A 60-year-old White male presented to the PH clinic for a second opinion. He had a history of prior tobacco use, mild obstructive pattern on pulmonary function testing with an FEV1 of 74%, and calcified granulomas with bulky mediastinal lymphadenopathy and fibrosing mediastinitis, presumed to be related to remote histoplasmosis infection with prior biopsy showing hyalinizing granuloma. Five years earlier, he had been evaluated by a different provider for New York Heart Association Functional Class (NYHA FC) 2 symptoms and echocardiographic PH. 6-minute walk test (6MWT) distance at that time was 384 m with no oxygen requirement. Contrasted computed tomography (CT) of the chest showed multiple large calcified pulmonary nodules with bulky calcified mediastinal adenopathy and mediastinal fibrosis causing severe stenosis of the distal right main pulmonary artery (PA) and mild stenosis of the bronchus intermedius and right middle and lower lobe bronchi (Fig. 1). Ventilation/perfusion (V/Q) scan demonstrated a marked right middle and right lower lobe perfusion defect (Fig. 2). They performed a right heart catheterization (RHC) that was consistent with precapillary PH with mean pulmonary artery pressure (mPAP) of 38 mmHg and PVR of 5.1 Wood Units (WU) (Table 1). He was monitored without starting any new therapies.

**Table 1:** Hemodynamic properties at baseline and after treatment.

	Case 1			Case 2	
Time	Baseline	Repeat evaluation 4 years later (no treatment)	Follow up after 1 year on treatment	Baseline	Follow up after 1 year on treatment
**mRAP (mmHg)**	3	10	4	16	8
**mPAP (mmHg)**	38	54	32	44	26
**PAWP (mmHg)**	10	15	10	15	9
**CO (L/min)**	5.43	3.86	6.15	6.58	6.53
**PVR (WU)**	5.1	10.1	3.6	4.4	2.6

At the time of our evaluation, he had progressed to NYHA FC 3. Echocardiography revealed normal left ventricular (LV) function, a mildly dilated right ventricle (RV) with normal function, and an estimated RV systolic pressure (RVSP) of 58 mmHg. He walked 274 m during a 6MWT and required 4 L (L) of oxygen with ambulation. Repeat cross-sectional imaging of the chest was unchanged. Repeat RHC was performed, and it showed worsening of his precapillary PH with mPAP of 54 mmHg and PVR of 10.1 WU (Table 1). Vasoreactivity testing with inhaled nitric oxide was negative. He was diagnosed with group 5 severe PH in setting of FM. Different management approaches were discussed with the patient and he agreed on a trial of pulmonary artery vasodilator therapy with close monitoring. He was started on sildenafil 20mg three times daily, and inhaled treprostinil solution at 3 breaths four times daily (QID) with successful weekly titration by 3 breaths as tolerated to 12 breaths QID. Subsequently, the patient was switched from inhaled treprostinil solution 12 breaths QID to the equivalent dose of treprostinil dry powder inhaler (DPI) of 64mcg QID. The patient tolerated this regimen well with improved dyspnea to NYHA FC 2, 6 MWT distance of 305 m and 2 L oxygen requirement. Repeat RHC after roughly 1 year on therapy showed hemodynamic improvement with mPAP 32 mmHg and PVR down to 3.6 WU (Table 1).

## 3 Case 2

**Fig. 3:**
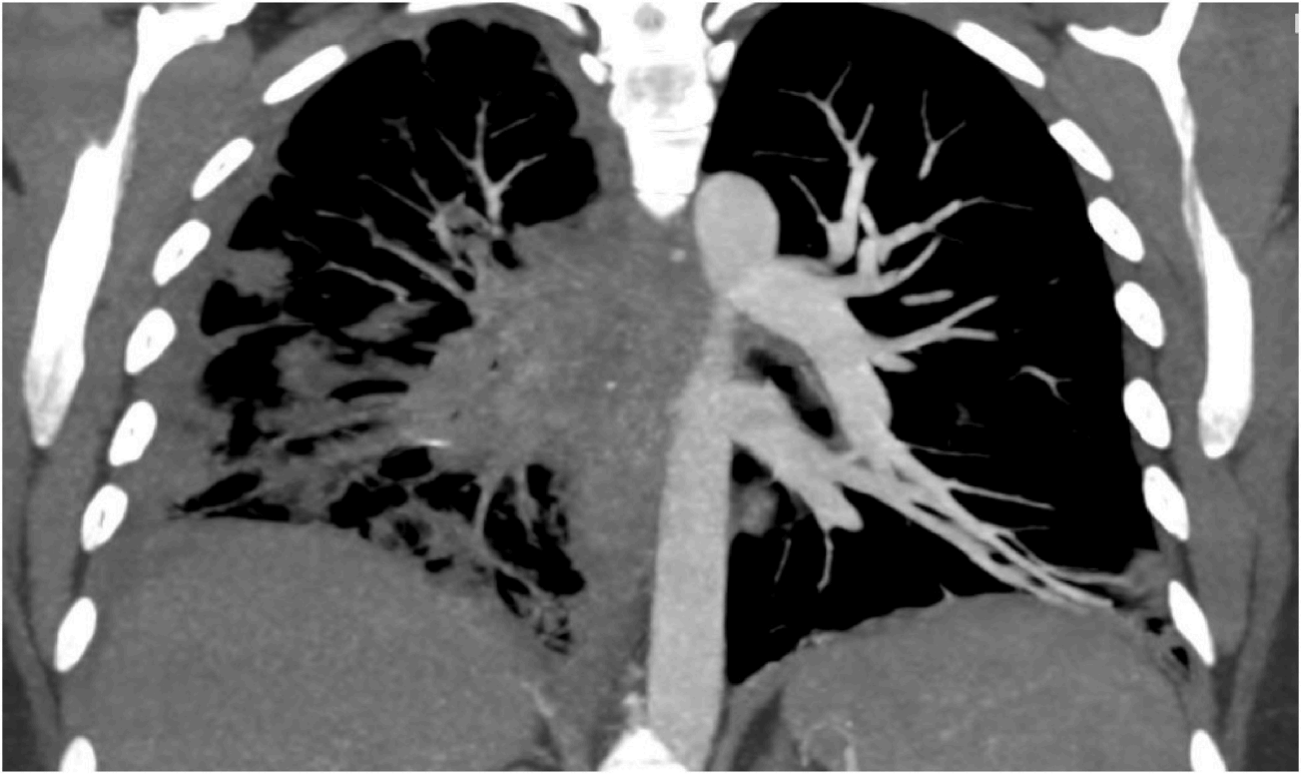
Coronal slice of computed tomography pulmonary angiography showing severe stenosis of the right pulmonary artery and mediastinal, hilar, and parenchymal fibrosis.

**Fig. 4:**
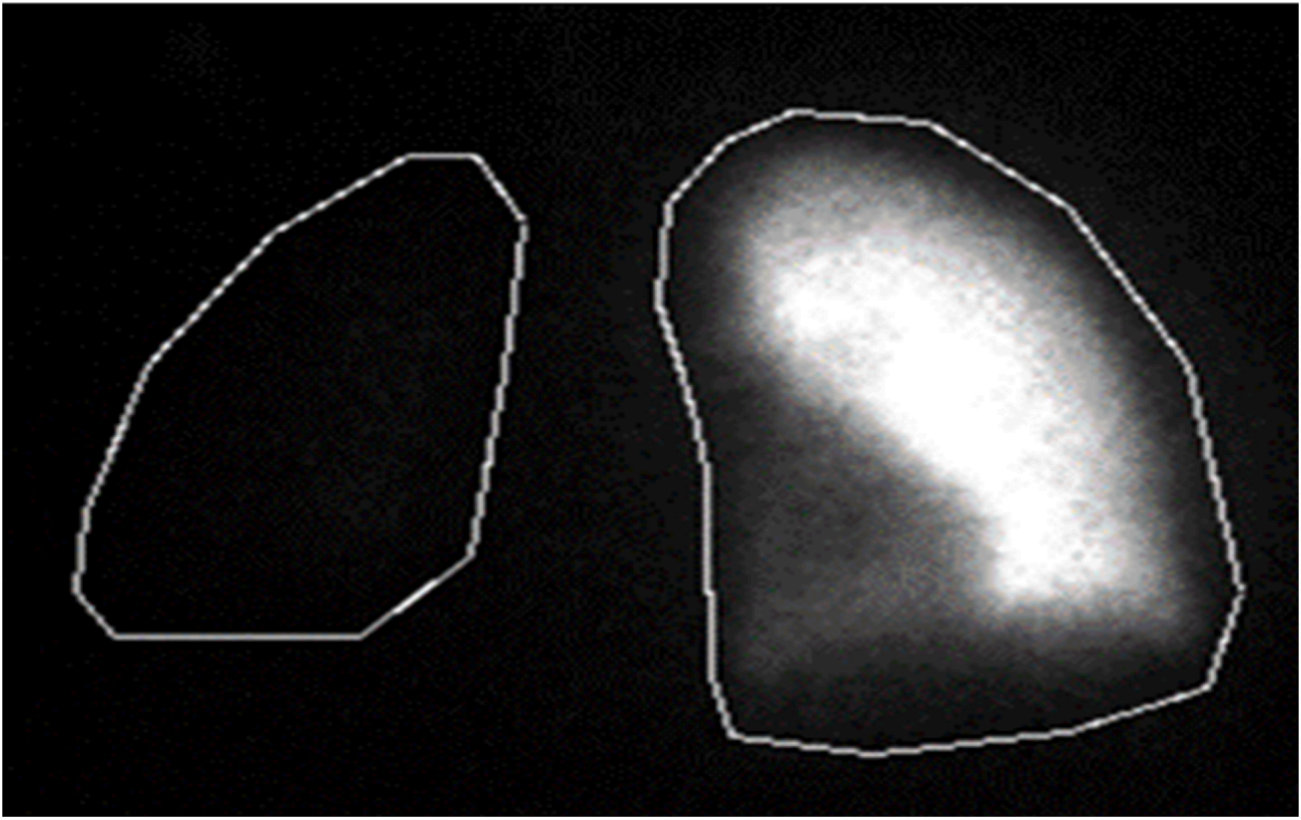
Left anterior oblique perfusion scan showing nearly absent perfusion to the right lung.

A 28-year-old White female with a history of idiopathic mediastinal fibrosis presented for evaluation of echocardiographic PH. Echocardiography showed normal left ventricular function with a moderately dilated and hypokinetic right ventricle and an elevated RVSP of 60 mmHg. Chest CT showed right mediastinal, hilar, and parenchymal fibrotic changes with near complete stenosis of the right sided pulmonary arterial system (Fig. 3). Perfusion scan estimated the right lung received only 3.5% of total pulmonary perfusion (Fig. 4). She had a 6-min walk distance of 341 m and was NYHA FC 3. Right heart catheterization was performed, and it showed precapillary pulmonary hypertension with mPAP 44 mmHg and PVR of 4.4 WU (Table 1). Vasoreactivity testing was performed using inhaled nitric oxide and it was positive with a decrease in mPAP to 33 mmHg and increase in CO to 8.12 L/min. Different management approaches were discussed with the patient and she agreed on a trial of high dose calcium channel blocker therapy with close monitoring. She was started on amlodipine with the dose titrated up to 30mg daily. She tolerated the titration well with subjective improvement in dyspnea to FC 2, normalization of her echocardiogram, and improvement of her 6-min walk distance to 407m without desaturation. Repeat RHC after roughly 1 year on therapy showed hemodynamic improvement with a mPAP of 26 mmHg, PVR of 2.6WU (Table 1). She was maintained on amlodipine 30 mg daily for 2 years with sustained clinical response but developed significant gingival hyperplasia, a known side effect of amlodipine, so she was transitioned to sildenafil 20 mg po three times per day with regression of the gingival hyperplasia. The patient maintained the clinical response on the phosphodiesterase 5 inhibitor.

## 4 Discussion

Fibrosing mediastinitis is a benign fibroproliferative disease characterized by mediastinal infiltration of a pauci-cellular hyperplastic fibrous tissue which infiltrates adipose tissue and often extrinsically compresses vital structures including large airways and thoracic vasculature [2]. It is a rare disorder with prevalence and etiology varying based on geography. Pathogenesis is incompletely understood but is believed to involve a dysregulated fibrogenic immune response to an inciting antigen [1]. In the United States, granulomatous response to infection with *Histoplasma capsulatum* is believed to be the most common provoking event, though numerous other etiologies have been suspected [1,2]. FM is variably progressive, and the disease course is highly heterogeneous [[2], [3], [4]]. Most patients present with respiratory symptoms [2]. Evaluation involves consideration of malignancy, assessment for the triggering etiology, and appraisal of anatomic involvement. This may include cross-sectional imaging, tissue biopsy, echocardiography, pulmonary function testing, endoscopy, and/or invasive hemodynamic assessment [1,4]. Unfortunately, treatment of the underlying etiology is often unsuccessful in altering disease course. There have been reports of successful management with immunosuppression in patients with evidence of active inflammation, though experience is limited [1,7]. Thus, management is typically focused on treating anatomic and physiologic sequela of this sclerosing disease [[1], [2], [3],^5^,^6^]. Though outcomes data is limited, FM is associated with significant morbidity and mortality, primarily from respiratory failure and/or cor pulmonale [[1], [2], [3]].

Pulmonary hypertension, currently defined as a mPAP >20 mmHg, is a well described complication of FM and contributes considerably to the disease's morbidity and mortality [1,4,8]. According to the most recent PH guidelines, PH-FM falls within group 5, ‘pulmonary hypertension with unclear and/or multifactorial mechanisms’ [8]. It can be categorized into 3 types based on vascular involvement with type I representing stenosis that involves mainly the pulmonary arteries (PAs) with possible adjacent bronchial involvement but without involvement of the pulmonary veins (PVs), type II which predominantly affect PVs, and type III which involves PAs, PVs, and bronchi [1].

Given the implications of untreated PH, providers that encounter patients with FM must maintain a high index of suspicion. The prevalence of PH within FM is not precisely known, but it appears that most cases of FM that come to clinical attention have some degree of PH [1]. Symptoms can include dyspnea, chest discomfort, exertional syncope and pre-syncope, lower extremity edema, and hemoptysis. Evaluation should proceed according to current PH guidelines and may include cross sectional imaging, echocardiography, V/Q scan, cardiac biomarkers, and invasive hemodynamics via RHC [8]. RHC can be technically difficult in PH-FM and should be performed by an experienced provider who is familiar with the case. An elevated PAWP can result from elevated left ventricular end-diastolic pressure (LVEDP), PV stenosis, or PA stenosis [8,9]. Reviewing chest imaging before the procedure is important to identify areas of stenosis to avoid inaccurate PAWP that may not reflect the true LVEDP. Depending on the suspected locations of involvement, measurements may need to be taken in multiple regions. Also, normal PAWP doesn't always rule out the possibility of pulmonary venous involvement, comparable to the hemodynamic profiles that are encountered in cases of pulmonary veno-occlusive disease. (1) Although guidelines recommend vasoreactivity testing only for idiopathic, heritable, and drug associated PAH, one of our patients had a positive vasoreactivity test with sustained clinical response with high dose calcium channel blocker treatment [8]. Finally, PH-FM can mimic chronic thromboembolic pulmonary hypertension on ventilation perfusion scans if cross-sectional imaging is not carefully reviewed. Failure to recognize these nuances can have meaningful implications on management.

Due to the infrequency and heterogeneity of the disease, there are no well-established treatment guidelines for PH-FM and management must be individually tailored. Oxygen should be provided to patients who meet qualifying criteria, and diuretics can be considered for those with evidence of hypervolemia [8]. Endovascular correction of pulmonary arterial or venous stenosis is an appealing strategy and has historically been the preferred approach. Unfortunately, due to the underlying disease process in PH-FM, these procedures carry an often unacceptably high complication and failure rate [1,5,10,11]. Surgical approaches have shown similarly poor outcomes [2,6]. Reports are limited, but as made evident in our cases, vasodilator therapy may improve both hemodynamics and patient symptoms and should carry significantly less risk than surgical or endovascular techniques when managed by an experienced team [1,4].

In our experience, selected patients with precapillary vascular phenotype can benefit from pulmonary artery vasodilators after informed discussion with the patient and with close monitoring of treatment response and possible side effects. Monitoring for any clinical worsening or development of pulmonary edema is prudent due to the possibility of PV involvement even with normal PAWP on RHC. Studies are needed to inform evidence-based treatment decisions for patients with precapillary PH-FM. Studying such a group of patients can be challenging due to the high complexity and disease heterogeneity.

## 5 Conclusion

Pulmonary hypertension associated with fibrosing mediastinitis (PH-FM) is a rare, complex, and heterogenous disease that carries significant morbidity. Management must be tailored to each case. Surgical and endovascular approaches have had disappointing results. In carefully selected patients, pulmonary vasodilator therapy may be beneficial, though more studies are required.

## Funding

There was no funding directly supporting the creation of this report.

## Declaration of competing interest

No conflict.

## References

[1] Wang A., Su H., Duan Y., Jiang K., Li Y., Deng M. (2022). Pulmonary hypertension caused by fibrosing mediastinitis. JACC Asia.

[2] Peikert T., Colby T.V., Midthun D.E., Pairolero P.C., Edell E.S., Schroeder D.R. (2011). Fibrosing mediastinitis: clinical presentation, therapeutic outcomes, and adaptive immune response. Medicine (Baltim.).

[3] Loyd J.E., Tillman B.F., Atkinson J.B., Des Prez R.M. (1988). Mediastinal fibrosis complicating histoplasmosis. Medicine (Baltim.).

[4] Seferian A., Steriade A., Jais X., Planche O., Savale L., Parent F. (2015). Pulmonary hypertension complicating fibrosing mediastinitis. Medicine (Baltim.).

[5] Ponamgi S.P., DeSimone C.V., Lenz C.J., Coylewright M., Asirvatham S.J., Holmes D.R. (2015). Catheter-based intervention for pulmonary vein stenosis due to fibrosing mediastinitis: the Mayo Clinic experience. Int. J. Cardiol. Heart Vasc..

[6] Mathisen D.J., Grillo H.C. (1992). Clinical manifestation of mediastinal fibrosis and histoplasmosis. Ann. Thorac. Surg..

[7] Westerly B.D., Johnson G.B., Maldonado F., Utz J.P., Specks U., Peikert T. (2014). Targeting B lymphocytes in progressive fibrosing mediastinitis. Am. J. Respir. Crit. Care Med..

[8] Humbert M., Kovacs G., Hoeper M.M., Badagliacca R., Berger R.M.F., Brida M. (2022). ESC/ERS Guidelines for the diagnosis and treatment of pulmonary hypertension. Eur. Heart J..

[9] Ingraham B.S., Packer D.L., Holmes D.R., Reddy Y.N.V. (2022). The hemodynamic spectrum of pulmonary vein stenosis from fibrosing mediastinitis. Cathet. Cardiovasc. Interv..

[10] Albers E.L., Pugh M.E., Hill K.D., Wang L., Loyd J.E., Doyle T.P. (2011). Percutaneous vascular stent implantation as treatment for central vascular obstruction due to fibrosing mediastinitis. Circulation.

[11] Fender E.A., Widmer R.J., Knavel Koepsel E.M., Welby J.P., Kern R., Peikert T. (2019). Catheter based treatments for fibrosing mediastinitis. Cathet. Cardiovasc. Interv..

